# The Importance of the Individual Baseline Immunometabolic and Cerebrovascular State in Correctly Interpreting Functional Neuroimaging Data: Lessons from Blood Mitochondrial Respirometry

**DOI:** 10.1159/nim/adxag004

**Published:** 2026-09-09

**Authors:** Alexander Karabatsiakis, Lisa Holper, Felix Scholkmann

**Affiliations:** a Biomolecular Stress and Trauma Research Group, Clinical Psychology II, Department of Psychology, University of Innsbruck, Innsbruck, Austria; b Department of Forensic Psychiatry and Psychotherapy, Psychiatric University Hospital Zurich, University of Zurich, Zurich, Switzerland; c Neurophotonics and Biosignal Processing Research Group, Biomedical Optics Research Laboratory, Department of Neonatology, University Hospital Zurich, University of Zurich, Zurich, Switzerland; d Neuroscience Center Zurich, University of Zurich and ETH Zurich, Zurich, Switzerland

**Keywords:** Immunometabolic disease, Functional neuroimaging, Mitochondrial function, Major depressive disorder, Functional tests, Biomarker research

Many acute and chronic diseases have an immunometabolic component. This applies not only to systemic conditions such as cardiovascular disease [1, 2], cancer [3], diabetes [4], multiple sclerosis (MS) [5], myalgic encephalomyelitis/chronic fatigue syndrome (ME/CFS) [6], and long-term effects of SARS-CoV-2 infection [7], but also to neuropsychiatric disorders, including autism spectrum disorder [8], bipolar disorder (BD) [9] and major depressive disorder (MDD) [10, 11].

Specific markers (biological or digital) are necessary for these diseases in order to enable a predictive, preventive, and personalized (3P) medical approach, ensuring optimal diagnosis, treatment adaptation, and therapeutic monitoring. From our perspective, functional *in vitro* and *in vivo* testing methods show particular promise in this regard. By “functional testing methods,” we mean evaluating how a subject’s physiology (e.g., the cardiorespiratory, central and autonomic nervous, immune, and endocrine systems) responds to a specific stimulus, and how these responses differ between patients and control subjects. We propose two categories of functional biomarker with substantial potential: (i) the mitochondrial bioenergetic state (see [12] for an introduction) and (ii) the neurovascular and neurometabolic state [13]. However, using these tests effectively as biomarkers requires careful consideration of a specific physiological aspect, which is detailed below and may also provide novel insights into the underlying pathophysiology of these diseases.

As a recent *Perspective* article in this journal explained [14], testing the mitochondrial functional state of immune cells in patient blood is an innovative approach. However, virtually all *in vitro* peripheral cellular respirometry tests are conducted exclusively at a standardized normothermic baseline of 37 °C. Although this methodology has already demonstrated altered mitochondrial function in MDD [15–17], it should be extended to include disorders with an immunometabolic component as these can be associated with abnormal body temperature. If these tests are performed at a temperature that differs from the subject’s actual body temperature, the results may be altered or require reinterpretation. For example, compared to healthy controls (HCs), depressed individuals have a significantly higher body temperature [18] and in specific brain regions [19], and elevated body temperature (see [20, 21] for methods) is generally associated with depressive symptoms [22]. An increase in body temperature has also been observed during manic episodes of BD [23] and relapsing-remitting multiple sclerosis (RMMS) [24]. Temperature anomalies have also been observed in patients with ME/CFS, with some studies showing decreased body temperature in winter [25] and others showing increased body and brain temperatures overall [26].

Therefore, to accurately assess mitochondrial function, we recommend cellular oxygen consumption rates are systematically evaluated across a physiological temperature range of 37–41 °C. Furthermore, when interpreting the results, consideration must be given to the subject’s core body temperature at the time the blood sample is obtained. In addition to temperature, pH is another important factor to consider, as noted [14]. Various methods of measuring core body temperature exist, each with their own respective advantages and disadvantages [20, 21, 27, 28]. Brain temperature can be measured non-invasively using techniques such as magnetic resonance spectroscopic imaging thermometry [29] and near-infrared spectroscopy [30]

It is incorrect to assume that that “background parameters” such as temperature and pH are identical in individuals with diseases and in healthy controls, as determined by functional methods (such as mitochondrial respirometry). To optimize *in vitro* functional tests of mitochondrial metabolism and facilitate the correct interpretation of the results, it is recommended that subject’s blood p*H* (easily determined, e.g., by blood gas analysis) and other relevant biochemical parameters such as lactate, partial pressure of O_2_ (PaO_2_), and CO_2_ (PaCO_2_), are measured.

Similarly, issues arises in functional neuroimaging, particularly in techniques that rely on hemodynamic signal changes, such as functional magnetic resonance imaging (fMRI) [31] and functional near-infrared spectroscopy (fNIRS) [32], when testing for abnormalities in brain activity in patients compared to HCs. It is often implicitly assumed that the physiological parameters of these groups are non-specific and identical. For example, it is problematic to assume that the baseline physiological state is similar and that only stimulus- or task-evoked activity differs. There is a complex relationship between the baseline state, the magnitude of activation-related change, and the measured signal contrast (activation minus baseline). Figure 1 illustrates this issue. Therefore, when planning and interpreting hemodynamic-based functional neuroimaging studies, we recommend using our “advanced model” (Fig. 1b) when planning and interpreting hemodynamic-based functional neuroimaging studies, rather than the “simple model” (Fig. 1a), which is still frequently used.

**Fig. 1.**
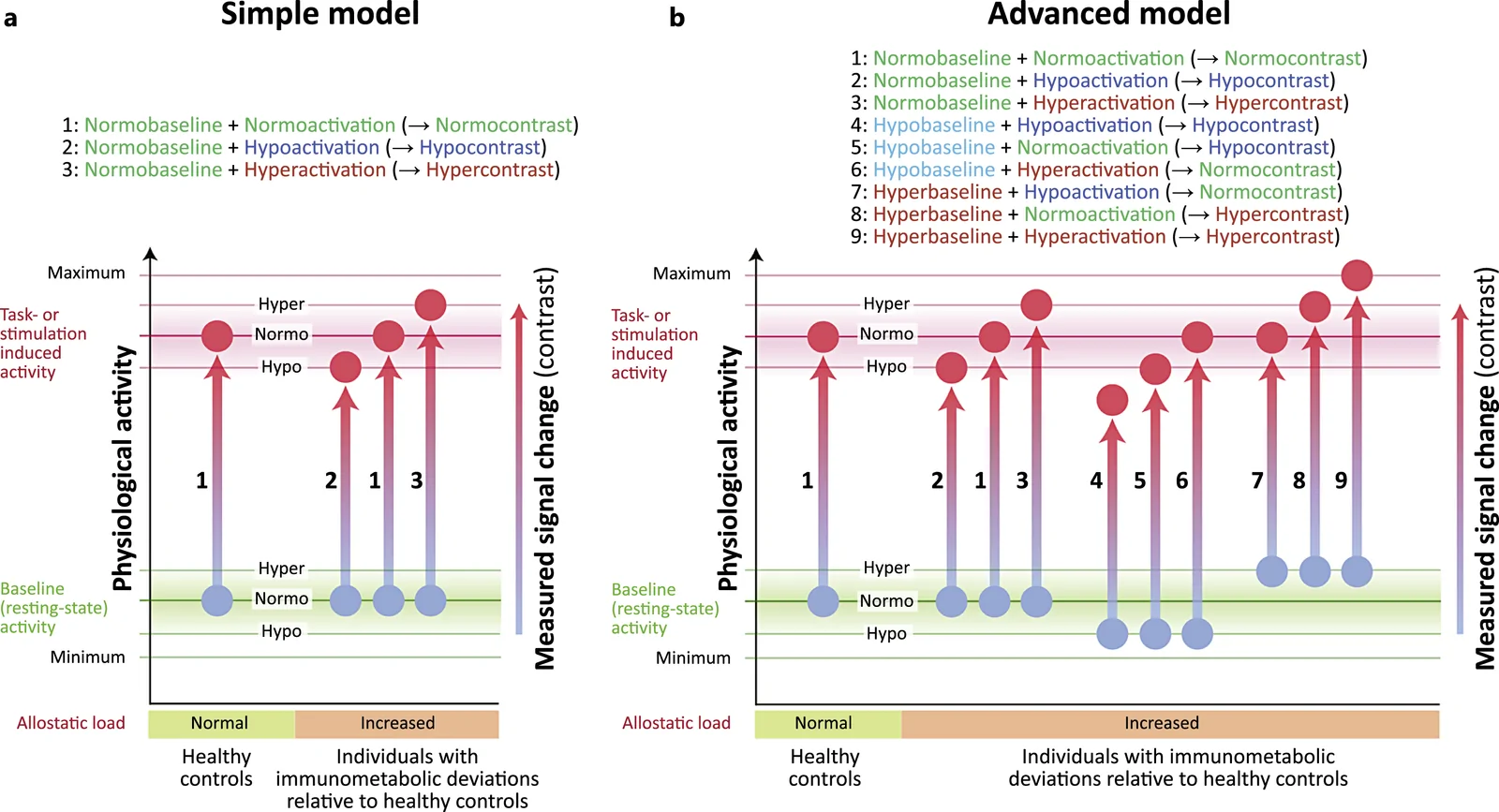
A comparison of two models that conceptualise physiological baseline states and induced activity in functional neuroimaging is presented below. **a** The conventional, oversimplified “simple model” assumes a uniform baseline (resting-state) of physiological activity across populations. In this framework, differences in the measured signal contrast (activation minus baseline) are directly attributed to alterations in task- or stimulation-induced activity (characterized as normo-, hypo- or hyperactivation), with potential variations in the underlying physiological baseline state being ignored. **b** The proposed “advanced model” accounts for a shifting physiological baseline. Under conditions of increased allostatic load (such as in individuals with immunometabolic deviations compared to healthy controls), baseline activity can shift independently, manifesting as hypo- or hyperbaseline. Consequently, as the baseline state and the magnitude of the activation change can vary independently, the measured signal contrast can no longer be definitively equated with the magnitude of the evoked activation change. This discrepancy can lead to statistical false positives, in which a different signal contrast emerges despite identical task-evoked activity magnitude, or false negatives, in which a genuine difference in task-evoked magnitude is masked and an identical signal contrast is observed between groups. Numbers 1–9 denote the distinct combinations of baseline states and activation types recommended to govern the final measured signal contrast.

We recommend adopting our advanced model whenever subject populations are being compared and it is expected that the allostatic load [33] will differ between them. Allostatic load is a general measure of physiological state that deviates from optimal health. Even when there is no difference in the magnitude of stimulus- or task-evoked activity between groups, the signal contrast can differ when the baseline activity differs from that of healthy controls (a statistical false positive). Conversely, differences in baseline physiology can mask genuine differences in stimulus- or task-evoked activity, resulting in similar signal contrast in both groups (a statistical false negative).

Another confounding factor in functional neuroimaging is neurovascular coupling (NVC), whereby brain activity directly triggers an increase in localized cerebral blood flow. Another factor is neurometabolic coupling, whereby localized brain activity triggers a corresponding increase in cellular energy metabolism. It may be flawed to assume that NVC and neurometabolic coupling function similarly in both HCs and patient cohorts with immunometabolic abnormalities. Indeed, a recent study investigating the relationship between BOLD fMRI and cerebral blood flow measured with arterial spin labeling demonstrated NVC alterations in MDD [34]. Furthermore, reduced cerebrovascular reactivity (CVR), i.e., reduced cerebral vasodilatory capacity, has been found in MDD [35–37]. As discussed in detail in the context of fNIRS neuroimaging [38], an abnormal CVR can affect fMRI measures, as unavoidable systemic physiological changes during the experiment can mask or mimic NVC-induced signal changes.

From a technical perspective, confounding effects in fMRI studies can be mitigated, at least partially, by: - employing calibrated fMRI (i.e., calculating CVR and correcting the fMRI signal accordingly) [39]; - using multimodal approaches that combine neuroimaging modalities (e.g. PET-EEG-fMRI [40]); or - including systemic physiological measurements (e.g., continuous non-invasive blood pressure, electrodermal activity, PaCO_2_, and cardiorespiratory activity) in the neuroimaging setup [41]. Furthermore, measuring the mmunometabolic state of the subject via blood analysis, including temperature-dependent mitochondrial metabolism, should be an integral part of future fMRI or fNIRS neuroimaging studies. Baseline serum lactate, which can be easily measured with point-of-care devices, may also be relevant. In addition, differences in baseline physiology may explain the inconsistent results often observed in fMRI and fNIRS studies. For example, task-based and resting-state fMRI results for MDD remain largely inconsistent [42].

In summary, assuming identical baseline physiology between HCs and individuals with immunometabolic diseases can lead to the misinterpretation of functional assays and neuroimaging results. Taking these baseline discrepancies into account in future functional investigations could substantially improve both fundamental pathophysiological research and subsequent biomarker development for immunometabolic disorders.

## Conflict of Interest Statement

Alexander Karabatsiakis was a member of the journal’s Editorial Board at the time of submission and is an Associate Editor of the Perspectives section. The remaining authors have no conflicts of interest to declare.

## Funding Sources

We acknowledge funding by the Swiss National Science Foundation (grant No. 184755). The funder had no role in the design, data collection, data analysis, and reporting of this study.

## Author Contributions

Alexander Karabatsiakis and Felix Scholkmann: writing – original draft, writing – review and editing, conceptualization, and visualization; Lisa Holper: writing – review and editing, project administration, resources, supervision, and funding acquisition.
